# Extrudability, printability, and strain rate sensitivity metrics of lightweight thermoplastic polyimide (PI) in material extrusion (MEX) additive manufacturing

**DOI:** 10.1039/d5ra05290d

**Published:** 2025-10-31

**Authors:** Nectarios Vidakis, Nikolaos Michailidis, Nikolaos Mountakis, Maria Spyridaki, Apostolos Argyros, Vasileios Stratiotou Efstratiadis, Emmanuel Stratakis, Markos Petousis

**Affiliations:** a Department of Mechanical Engineering, Hellenic Mediterranean University Heraklion 71410 Greece vidakis@hmu.gr mountakis@hmu.gr mspyridaki@hmu.gr markospetousis@hmu.gr +302810379227; b Physical Metallurgy Laboratory, Mechanical Engineering Department, School of Engineering, Aristotle University of Thessaloniki 54124 Thessaloniki Greece nmichail@auth.gr aargyros@auth.gr vstratio@meng.auth.gr; c Centre for Research & Development of Advanced Materials (CERDAM), Centre for Interdisciplinary Research and Innovation, Balkan Centre Building B', 10th km Thessaloniki-Thermi Road 57001 Thessaloniki Greece; d Foundation for Research and Technology-Hellas (FO.R.T.H) Heraklion Crete Greece stratak@iesl.forth.gr; e Qingdao Innovation and Development Center, Harbin Engineering University Qingdao China

## Abstract

Among the high-performance polymers (HPPs) deployed in material extrusion 3D printing, thermoplastic polyimide (PI or TPI) dominates in terms of chemical stability and thermomechanical performance. Such features, suitable for challenging operational environments, nationalize and defend the remarkably expensive filament market. This study comprehensively explores the physical, thermal, rheological, key mechanical, and strain rate sensitivity metrics of PI. The PI pellets were melt-extruded into filaments under optimized thermomechanical control settings. A detailed experimental course, including elemental and chemical characterization, scanning electron microscopy morphological assessments, and Raman spectroscopy, was implemented. Thermal stability and phase transitions were determined using differential scanning calorimetry and thermogravimetric analysis. The rheological response was determined through viscosity/stress tests and melt flow rate measurements at various temperatures. A dynamic mechanical analysis was performed. Moreover, standard quasi-static mechanical tests documented the tensile, bending, impact, and microhardness performance of 3D printed specimens. Finally, the strain rate sensitivity metrics of the PI were derived from forty-five 3D printed tensile specimens subjected to nine steps with elongation speeds ranging from 10 to 300 mm min^−1^. Remarkably, the sample tested at 25 mm min^−1^ exhibited optimal mechanical performance, whereas a superior toughness was observed at 300 mm min^−1^. The strain rate sensitivity index and various other rate-dependent interactions were determined and comprehensively discussed. The inclusive findings herein provide critical insights into the overall performance of thermoplastic polyimide in additive manufacturing, aiming to support its broader exploitation in advanced engineering applications.

## Abstract 2

Strain rate sensitivity of MEX 3D printed polyimide (PI) parts.

## Graphical abstract

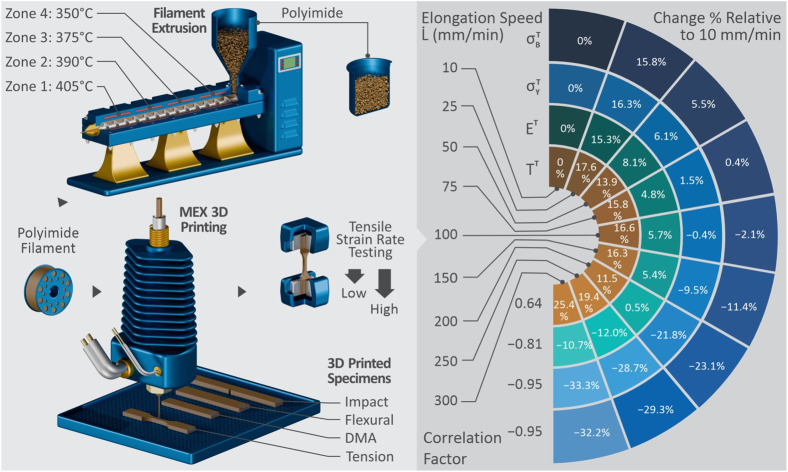

## 1. Introduction

Additive manufacturing (AM) technologies offer significant advantages, including the ability to produce parts with complex geometries^1^ and structures at reduced costs^2^ while minimizing material waste.^3^ These benefits have propelled AM into diverse fields,^4^ each with unique application requirements, spanning the biomedical,^5–7^ automotive,^8,9^ aerospace,^10,11^ electronics,^12,13^ defence,^14,15^ and construction^16–18^ sectors.

Extensive market analyses^19–21^ highlight the rapid expansion of the AM industry. For instance, Grand View Research projects a compound annual growth rate (CAGR) of 23.3% from USD 20.37 billion in 2023 through 2030.^22^ Similarly, Fact.MR forecasts growth from USD 26.79 billion for the year 2024 to USD 189.34 billion by the year 2034, achieving a CAGR of 21.6%,^23^ while Data Bridge anticipates an increase from USD 91.84 billion in the year 2024 to USD 419.22 billion by the year 2032, reflecting a CAGR of 20.90%.^24^ These projections underscore the transformative economic impact and broad adoption of AM technologies.

A wide range of materials, including polymers,^25,26^ ceramics,^27^ and metals, are available for diverse applications.^28^ Among these, polymers are the most commonly used, favoring their versatility and variety. Commonly used polymers in material extrusion (MEX) 3D printing include Polylactic Acid (PLA),^29^ Acrylic Styrene Acrylonitrile (ASA),^30^ Acrylonitrile Butadiene Styrene (ABS),^31^ high-density polyethylene (HDPE),^32^ polycarbonate (PC),^33^ and poly(methyl methacrylate) (PMMA),^34^ which are utilized in pure form or as matrices in composites. They are generally classified into engineering,^35^ standard,^36^ and high-performance polymers (HPP),^37,38^ based on their properties and intended applications. One polymeric material is considered to be an HPP if it satisfies certain conditions regarding its durability, the temperature at which it thermally decomposes, the rate at which it loses weight, its glass transition temperature, and its overall mechanical properties.^39^ Notably, high-performance polymers remain underexplored in the context of additive manufacturing, with the current literature revealing significant gaps in the understanding of their behavior and performance when 3D printed.

HPP referenced in additive manufacturing research^40^ includes notable materials, such as polyetheretherketone (PEEK),^41–44^ polyetherimide (PEI),^45–47^ polyphenylenesulfone (PPSU),^48–50^ polyimide (PI),^51,52^ and polyvinylidene fluoride (PVDF).^53–55^ These polymers have found applications across a wide spectrum of industries,^56^ including medical devices,^57–59^ aerospace,^60,61^ space technology,^62^ as well as the energy, transportation, and defense sectors.^36^ Their exceptional thermal and mechanical properties, along with their outstanding stability under extreme conditions, underpin their growing preference for high-performance applications.^63^ Their high specific strength (strength-to-weight ratio) enables the production of components that are not only mechanically robust, but also significantly lighter than their metal counterparts.^64^ Moreover, the ability to fabricate complex geometries through MEX 3D printing further enhances the weight efficiency of HPP parts by enabling the design of lattice- or topology-optimized structures without compromising structural integrity. For this reason, the mechanical properties of HPP, such as PEEK^65^ and PEI,^66^ have been reported, along with studies on the optimization of their quality characteristics as a function of the applied 3D printing settings.^67^

Polyimides (PI) belong to a class of aromatic polymers (repeating aromatic units connected with imide linkages) characterized by imide functional groups (–CO–NH–CO–). Its molecular architecture features robust aromatic backbones, characterized by strong intermolecular interactions (π–π stacking and hydrogen bonding). Their remarkable thermal and oxidative stability, mechanical strength, and chemical resistance are attributed to these structural features.^68,69^ Thermoplastic PI, in contrast to its thermoset counterpart, possesses linear, non-crosslinked structures, allowing it to melt, which is necessary for use in MEX 3D printing processes.

PI specifications categorize it as an Ultra High Performance Polymer (UPP), with a glass transition temperature (*T*_g_) of 297 °C (a common thermoplastic such as PLA has a *T*_g_ of approximately 60 °C (ref. 70)), initial decomposition temperature (IDT) of 573 °C (a common thermoplastic such as PLA has an IDT of approximately 320 °C (ref. 71)), ultimate tensile strength (UTS) of 86 MPa (a common thermoplastic such as PLA has a UTS of approximately 45 MPa (ref. 71)) and Young's modulus of 3 GPa (ref. 40, 72 and 73) (a common thermoplastic, such as PLA, has a Young's modulus of 0.8–2.5 GPa (ref. 74)).

UPP, such as PI, has notable advantages over common polymers, in addition to their higher mechanical strength. They feature higher thermal stability and chemical resistance, which makes them suitable for functional components fabrication in applications such as automotive, aerospace, energy, defense, and electronics,^75^ domains where conventional polymers are inadequate. PI is also used in lightweight structures^76^ in the engineering field and in electronics for flexible antennas^77^ and stretchable electronics^78^ manufacturing. Thus, the industrial merit of using PI in the fabrication of components is high.

Despite these advantages, the use of high-performance polymers in MEX printing presents significant challenges. They require high temperatures for processing and specialized equipment. Their high costs are also a dissuasive factor in their use. During 3D printing, warping is often an issue (affecting the dimensional accuracy of printed components), and poor interlayer adhesion can also negatively affect the mechanical response of parts built with HP polymers using the MEX method.

In particular, PI is distinguished by its superior mechanical strength,^79^ excellent thermal stability,^80^ and resistance to solvents, irradiation, impact, corrosion, fatigue, abrasion, and high temperatures, in addition to its electrical insulating capabilities.^81,82^ PI has been widely utilized in demanding sectors, such as space exploration,^83^ automotive industries, and microelectronics.^84^ In these domains, PI components include honeycomb structures and self-lubricating parts,^85,86^ and recent studies have highlighted its promising role in lithium-ion battery technology.^87^

In addition to standalone applications, PI has been incorporated into composite materials^84,88^ designed to meet mechanical, thermal, and electrical insulation requirements.^89^ Additionally, PI-based composites have specialized functions such as thermally conductive polymers,^90^ gas separation membranes,^91^ space-grade materials,^92^ and electrodes for electrocatalysis and sensing technologies.^93,94^ Moreover, PI's biocompatibility of PI has prompted its exploration in fused filament fabrication (FFF) processes, broadening its potential use in biomedical applications.^95^ In 3D printing, the 3D printing parameters that affect the mechanical strength, strain, water absorption, electrical conductivity, and porosity of the parts were investigated.^96–98^

Market analyses^99–101^ provide valuable insights for expanding the polyimide (PI) industry across various forecast periods. For instance, Zion Market Research reported a PI market size of USD 7.5 billion in 2022, projecting growth to USD 12.1 billion by 2030 with a compound annual growth rate (CAGR) of 6.1%.^102^ Similarly, Mordor Intelligence estimated the PI market at USD 5.46 billion in the year 2024, expecting it to reach USD 7.6 billion by the year 2029, reflecting a CAGR of 6.83%.^103^ Additionally, Grand View Research has highlighted a market size of USD 2.31 billion in 2022, with a forecasted CAGR of 7.8% between 2023 and 2030.^104^ These reports collectively underscore the robust and steady growth anticipated for PI in the coming decade.

The present study aims to enrich the existing body of literature on PI by investigating its 3D printing potential, thermal characteristics, rheological behavior, and mechanical performance under a spectrum of strain rates. Strain rate plays a crucial role in determining the mechanical response of polymeric materials processed *via* material extrusion (MEX) 3D printing. Previous research on common engineering polymers, such as polyethylene terephthalate glycol (PETG),^105^ polyamide 12 (PA12),^106^ PLA, polypropylene (PP), ABS,^107^ PC,^108^ PMMA, and thermoplastic polyurethane (TPU),^109^ revealed variations in their responses under different strain rates when subjected to tensile or compressive uniaxial forces. UPP, such as PI, exhibit strain rate-dependent properties owing to their viscoelastic and semicrystalline or amorphous molecular structures.^110^ The importance of the strain rate metric in the mechanical performance of 3D printed parts made with HPP has been previously highlighted for PSU and PPSU HPP.^110^ A literature review revealed no similar research on PI polymers.

Furthermore, in 3D printed parts, the layer-by-layer architecture introduces anisotropy and stress concentration zones that can amplify the strain rate effects during high-strain-rate loading.^111^ Understanding the strain rate behavior of UPP is vital for the accurate prediction of part performance in real-world applications, where loading can have a stochastic nature. Thus, the strain rate is a fundamental variable for the integrity and reliability of UPP components produced by MEX 3D printing. This is more crucial in HPP and UPP because of their specifications, and they operate in harsh working environments.^112^

This work provides insights into the strain-rate-dependent mechanical response of MEX 3D printed PI high-performance polymers by performing carefully executed tensile testing at various elongation rates. This work presents a thorough understanding of how tensile properties fluctuate with an increase in strain by revealing the viscoelastic and strain-hardening behavior of the PI high-performance thermoplastic material created through additive manufacturing. The mechanical response to increasing strain rates is compared and correlated with the microstructural features of the thermoplastic, applying the understanding of the isotropic properties of the 3D printed parts to understand the strain-dependent deformation and failure mechanisms. These investigations provide significant information when designing MEX AM components that use PI HPP, as variable loading conditions are common in real-world applications.

Tensile tests were conducted at elongation speeds ranging from 10 to 300 mm min^−1^ (10, 25, 50, 75, 100, 150, 200, 250, and 300 mm min^−1^) to evaluate the key mechanical properties, including yield strength, ultimate tensile strength, tensile modulus, and tensile toughness. The PI raw material was first extruded into a filament form, which was then utilized for the 3D printing of the test specimens. The extrudability of these materials was also tested. Comprehensive thermo-analytical analyses were performed using thermogravimetric analysis (TGA) and differential scanning calorimetry (DSC), and the rheological properties were examined through viscosity and stress curves along with melt flow rate (MFR) measurements. Dynamic mechanical analysis (DMA) further revealed the loss modulus, storage modulus, and damping factor across relevant temperature ranges (response under combined dynamic and thermal loading). Among the tested samples, the specimen tested at 25 mm min^−1^ exhibited superior overall mechanical properties, with the notable exception of tensile toughness, which peaked in the sample tested at the highest elongation speed of 300 mm min^−1^. The current research covers a gap in the literature, providing a full assessment of PI UPP properties within the context of MEX 3D printing. It is also a premium guide for the extrudability and printability of PI UPP, as these were thoroughly examined and optimized for this research.

## 2. Materials and methods

**Fig. 1:**
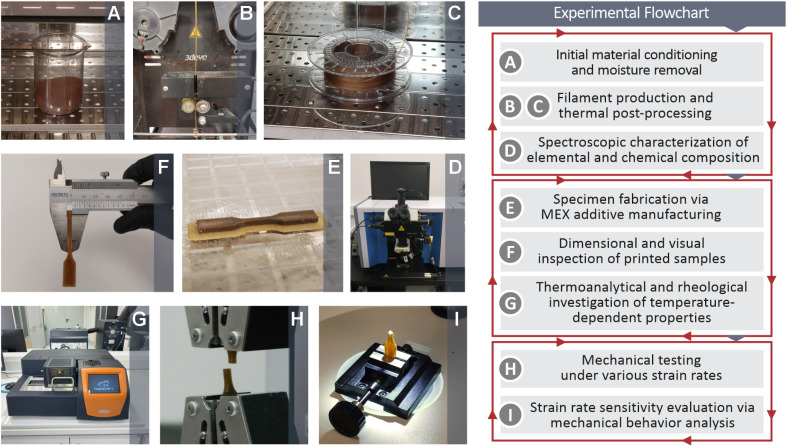
Pictures revealing the procedures of this research (left side) and the respective experimental flowchart (right side): (A) PI material conditioning and removal of the existing moisture, (B) and (C) filament extrusion and thermal post-processing, (D) elemental and chemical composition through stereoscopic characterization, (E) MEX AM specimen printing, (F) dimension measuring and visual inspection of the specimens, (G) thermo-analytical and rheological investigation of the properties depended from temperature, (H) mechanical performance examination through testing under different strain rates, (I) analysis of the mechanical behavior aiming strain rate sensitivity evaluation.

The left side of Fig. 1 shows images captured during the main processes of this study, which are also mentioned in the experimental flowchart on the right side of Fig. 1. First, the PI raw material was prepared and allowed to dehydrate (Fig. 1A) before filament extrusion and thermal post processing (Fig. 1B and C). Spectroscopic characterizations of both elemental and chemical compositions are shown in Fig. 1D. Next, MEX 3D printing (AM) was used to fabricate specimens (Fig. 1E). Their dimensional and visual inspections were performed (Fig. 1F). The samples were then subjected to thermoanalytical and rheological analyses (Fig. 1G). 3D printed coupon strain rate mechanical testing was conducted (Fig. 1H), and the strain rate sensitivity was evaluated by analyzing the mechanical behavior of the samples (Fig. 1I).

### 2.1. Material

The PI material (also known as TPI, *i.e.*, thermoplastic PI) was obtained in pellet form from Mitsui Chemicals (Tokyo, Japan). The grade was AURUM PL450C. The main specifications of the grade according to the information provided in the datasheet††https://jp.mitsuichemicals.com/content/dam/mitsuichemicals/sites/mci/documents/sites/default/files/media/document/2018/pl450c_0.pdf.coredownload.inline.pdf, accessed 28/06/2025. are provided in Table 1.

**Table 1:** PI raw material specifications (AURUM PL450C)

Specific gravity	1.33	Elongation	90% (ASTM D638)
Tensile strength	92 MPa (ASTM D638)	Flexural strength	137 MPa (ASTM D790)
Izod impact strength	88 J m^−1^ (ASTM D256)	Rockwell hardness (*R* scale)	129 (ASTM D785)
Heat distortion temperature	230 °C (ASTM D648)		

### 2.2. Preparation and extrusion of the filament

The filament was extruded using a desktop extruder model named Precision 450 (3D Evo, located in Utrecht, Netherlands), following the extruding conditions and specifications based on the polymer characteristics according to the manufacturer, as well as preliminary tests. The diameter of the filaments was 1.75 mm, as suggested for 3D printing of the coupons. The heating zones 1 to 4 of the extruder, beginning from the nozzle and ending at the hopper, had temperatures of 405 °C, 390 °C, 375 °C, and 350 °C, respectively. The speed of the screw was adjusted to 7 rpm, and the cooling speed of the fan was adjusted to 30%.

### 2.3. PI thermoplastic characterization: Raman, rheology, morphological, thermal

The characterization techniques listed herein were implemented within the context of this study to reveal the thermoplastic properties of PI. The specifications and methodology applied for each technique are presented in detail in the SI.

• A LabRAM HR Raman spectrometer (HORIBA Scientific, Kyoto, Japan) was used to acquire the Raman spectra.

• A DHR-20 rotational rheometer (Discovery Hybrid Rotational Rheometer, TA Instruments, New Castle, Delaware, United States) was used for the rheological and thermomechanical examinations (viscosity and DMA).

• Morphological evaluation: energy-dispersive X-ray spectroscopy (EDS) was performed using a field-emission Scanning Electron Microscopy (SEM) apparatus (JSM-IT700HR, Jeol Ltd, Tokyo, Japan). SEM illustrations for morphological evaluation were acquired with the same apparatus (mode: high-vacuum, 20 kV, Au sputtering of PI 3D printed coupons).

• Thermogravimetric Analysis (TGA) was performed using a Discovery Simultaneous Thermal Analyzer SDT 650 (TA Instruments, New Castle, Delaware, United States).

• Endothermic and exothermic (Differential Scanning Calorimetry – DSC) were both conducted with the assistance of the apparatus Discovery-Series DSC 25 (manufactured by TA Instruments, located in New Castle, Delaware, United States).

### 2.4. Coupons 3D printing

To manufacture the 3D printed coupons, a 3D Gence F421 3D printer (Przyszowice, Poland) was utilized, and V-type samples with 3.2 mm thickness were produced following the D638-02a standard of the American Society for Testing and Materials (ASTM). Nine tensile speeds were assessed, each with five replicas constituting forty-five PI coupons created by applying the same settings. Nozzle temperature was 420 °C, build plate temperature was 150 °C, chamber temperature was 70 °C, nozzle diameter was 0.4 mm, printing speed was 30 mm s^−1^, layer height was 0.2 mm, infill density was 100%, infill pattern was rectilinear (±45°, orientation was shifted by 90 °C between successive layers to reduce anisotropy in the samples), there were two perimeters (outer walls) and part cooling fan was 0% (disabled). It should be noted that prior to printing, the filament was dried in a convection oven at 120 °C for 8 h, in accordance with standard recommendations for polyimide-based materials.

### 2.5. Mechanical testing

The objective of this study was to assess the impact of the speed at which a load is applied (strain rate) on the performance of a UPP PI thermoplastic and report its response. For completeness, mechanical tests were carried out according to the respective standards for uniaxial (tensile, ASTM D638), flexural (three-point-bending, ASTM D790), impact (Charpy, notched, ASTM D6110) loadings, and microhardness (M-H, ASTM D384). In addition to the static loading tests, Dynamic Mechanical Analysis (DMA) was performed to evaluate the response of the PI thermoplastic under combined dynamic mechanical (three-point bending) and thermal loading. The conditions are presented in the SI.

The apparatus used for tensile testing with different strain rates of the PI 3D printed coupons was a model named MX2 (by the company IMADA, located in IL, USA). Standardized grips were used for testing. The tensile speeds applied to the samples were 10 (compatible with the ASTM D638 standard for static loading), 25, 50, 75, 100, 150, 200, 250, and 300 mm min^−1^ (at room temperature). These strain rates were selected to cover a broad range of operating conditions, from low to high strain rates. The elongation and strain rates are presented in Table 2.

**Table 2:** Strain rate and elongation speed

Speed of elongation (mm min^−1^)	Strain rate (s^−1^)	Speed of elongation (mm min^−1^)	Strain rate (s^−1^)
10	0.011	150	0.167
25	0.028	200	0.222
50	0.056	250	0.278
75	0.083	300	0.333
100	0.111		

The following formulas were considered for the calculation of the sizes utilized herein:1
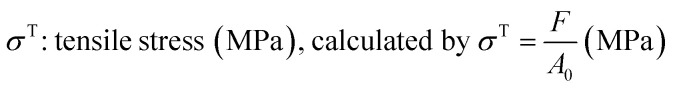
where *F*: applied tensile force (N) and *A*_0_: nominal cross-section area (mm^2^).2
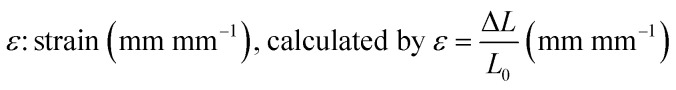
where Δ*L*: specimen elongation (mm) and *L*_0_: initial specimen length (mm) and *L*_0_ = 15 (mm).

3 *σ*^T^_B_: tensile ultimate strength (MPa) is found by the formula *σ*^T^_B_ = max(*σ*^T^) (MPa)

*L̇*: elongation speed (mm min^−1^) is: *L̇* = {10, 25, 50, 75, 100, 150, 200, 250, 300} (mm min^−1^).

*E* ^T^: tensile modulus of elasticity (MPa), calculated according to the ASTM D638 standard.4
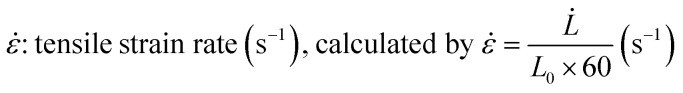
5
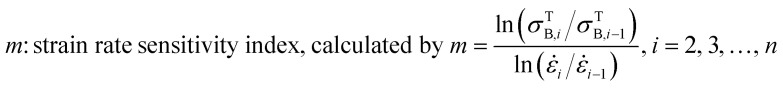

6 Where *σ*^T^_B,*i*_, *σ*^T^_B,*i*−1_: tensile ultimate strength values for two successive strain rates (MPa) and *ε̇*_*i*_,*ε̇*_*i*−1_: strain rates corresponding to the tensile ultimate strength values *σ*^T^_B,*i*_ and *σ*^T^_B,*i*−1_ (s^−1^) and *n*: number of tested elongation speeds is *n* = #(*L̇*)

The formula for the calculation of *T*^T^: tensile toughness (MJ m^−3^) is:7
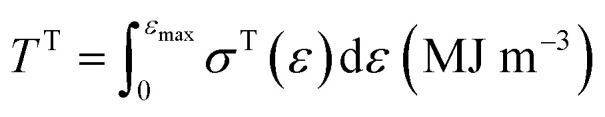

8 Where *ε*_max_: maximum strain (mm mm^−1^) is calculated by *ε*_max_ = max(*ε*) (mm mm^−1^)

*ε̇*_max_ (m): strain rate at maximum strain rate sensitivity index *m* (s^−1^), calculated by

9 *ε̇*_max_ (m) = *ε̇*_*i*_, where *m*_*i*_ = max{*m*_1_, *m*_2_, …, *m*_*n*_}, *i* = 1, 2, …, *n* (s^−1^)

Where *m*_max_: maximum strain rate sensitivity index, which is

10 *m*_max_ = max{*m*_1_, *m*_2_, …, *m*_*n*_}, *i* = 1, 2, …, *n*

## 3. Results

### 3.1. Raman and EDS spectroscopy

**Fig. 2:**
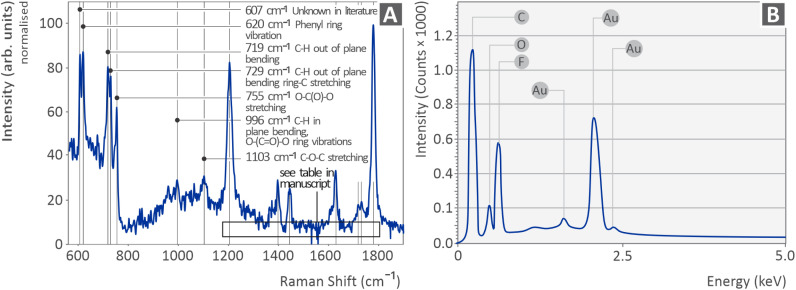
(A) Raman spectroscopy results and (B) EDS results indicating the chemical elements of the sample investigated.

In Fig. 2 (left side), the Raman spectrum of the pure PI is shown in Fig. 2 (left side). Table 3 lists the Raman peaks acquired from the PI coupon (pure material), which were obtained from the literature, together with their respective references. Furthermore, Fig. 2 (right side) displays the results of the EDS analysis, showing the intensity of the detected elements after analyzing the samples (the Au shown is from the coating of the samples to avoid charging). EDS was used to determine the composition of the material. In addition to C and O, which are common elements in polymeric materials, fluorine (F) was also detected, which is also an expected element in PI HPP.^113^ Gold was found because of the sputtering of the samples prior to capturing the SEM images. The samples were gold-coated to avoid charging issues. Thus, the EDS findings verified the absence of any peculiar or unexpected elements in the composition of the PI samples. Therefore, raw PI material was used to fabricate the coupons for testing.

**Table 3:** Raman (significant) peaks and their assignments from the PI UPP

Wavenumber (cm^−1^)	Intensity	Assignment of the Raman peaks
607	Strong	Unknown in the literature
620	Strong	Phenyl ring vibration^114^
719	Strong	C–H out-of-plane bending^114^
729	Strong	C–H out-of-plane bending;^114^ ring-C stretching^115^
755	Strong	O–C(O)–O stretching^116^
996	Medium	C–H in plane bending;^114^ O–(C=O)–O ring vibrations^115^
1103	Medium	C–O–C stretching^114^
1205	Strong	C–O–C stretching^116^
1398	Medium	C–H_3_ deformation^114^
1445	Medium	C–H_3_ deformation;^114,115,117^ C–H_2_ deformation;^114,115^ C–H_3_ symmetric bending^114,116,118^
1630	Medium	Phenyl ring stretch^116^
1718	Weak	C=O bond;^117,119^ C–O–C symmetric stretching^120^
1731	Weak	C=O bond;^117,119^ C–O–C symmetric stretching^120^
1779	Very strong	C=O stretching^115,116,118^

The Raman spectra obtained for the polyimide (PI) sample revealed a rich set of vibrational features that are characteristic of the molecular structure of the polymer. The spectral profile is consistent with the literature for aromatic polyimides, confirming the reliability of the measurements. The most prominent peaks in the spectrum are observed at 620, 719, 729, 755, 1205, 1445, and 1779 cm^−1^, each corresponding to a well-established vibrational mode within the PI backbone. The bands at 620 cm^−1^ and 1630 cm^−1^ are attributed to phenyl ring vibrations and stretching, respectively, which reflect the aromatic nature of the polymer. The strong peaks at 719 and 729 cm^−1^ are assigned to C–H out-of-plane bending, while the 755 cm^−1^ band is associated with O–C(O)–O stretching, defined in the imide group. The intense 1779 cm^−1^ band was indicative of C=O bond stretching, confirming the presence of imide. Noticeably, a particularly strong and distinct Raman band is observed at 607 cm^−1^. However, it is not feasible to identify a clear assignment in the literature. Its intensity and proximity to the 620 cm^−1^ phenyl-ring vibration suggest that it may originate from a similar structural mode unique to the specific PI material studied here. Raman spectroscopy is particularly valuable for identifying specific functional groups, monitoring crystallinity, and detecting structural changes induced by processing, temperature, or mechanical stress. In this study, was employed for completeness and verification of the PI HPP structure. In all test cases, the same PI samples were used; therefore, no changes were expected in their structures between the test cases.

### 3.2. Thermal properties of PI filament

**Fig. 3:**
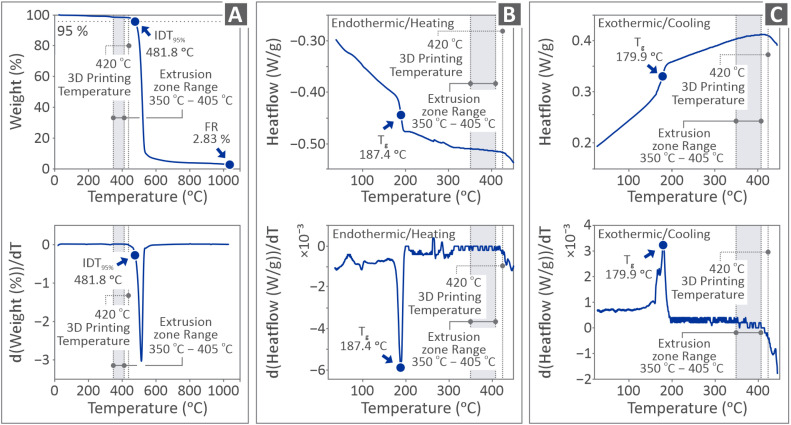
PI filament-related results from the investigation of their thermal properties, including curves for (A) TGA, (B) endothermic/heating behavior (DSC), and (C) exothermic/cooling behavior (DSC). The bottom graphs in each figure show the respective derivatives of the top graphs (*T*_g_: glass transition temperature (°C), IDT@95%: initial decomposition temperature at 95% of the original mass (°C)).

Fig. 3 shows the results derived from the thermal examination of the PI filament samples, that is, the TGA curves (Fig. 3A), DSC curves for the endothermic/heating behavior (Fig. 3B), and exothermic/cooling behavior (Fig. 3C). In the TGA graphs, the initial decomposition temperature (IDT) of 95% and the final residue (FR) are highlighted. In the graph, the 3D printing temperature and extrusion zone temperature ranges are depicted as well, compared with the thermal properties of the PI HPP. The *T*_g_, 3D printing, and extrusion zone temperature ranges are depicted in the DSC graphs. The bottom row of the figure shows the derivative of the top graph.

PI exhibited a thermal decomposition profile in close agreement with the literature values, as shown in Fig. 3A.^121^ A significant weight loss (*T*_idt_) was observed at 481.8 °C, reflecting its inherent thermal stability. The PI demonstrated a single-step degradation process, which is indicative of a well-defined and homogeneous structure.^122^ Finally, the residual mass of 2.83% was attributed to the formation of a thermally stable residue. This also highlights the material's resistance to complete volatilization at elevated temperatures, reinforcing its suitability for high-temperature applications. The derivative (Fig. 3A bottom graph) shows no significant incidents, in addition to weight loss due to decomposition. The glass transition temperature (°C) (*T*_g_) of PI was thoroughly analyzed using DSC, both during the heating and cooling cycles, as illustrated in Fig. 3B and C. During the heating cycle (Fig. 3B), *T*_g_ can be seen at 187.4 °C, as indicated by the heat flow curve and the corresponding peak in the derivative plot (Fig. 3B bottom graph).

### 3.3. Viscosity and MFR

**Fig. 4:**
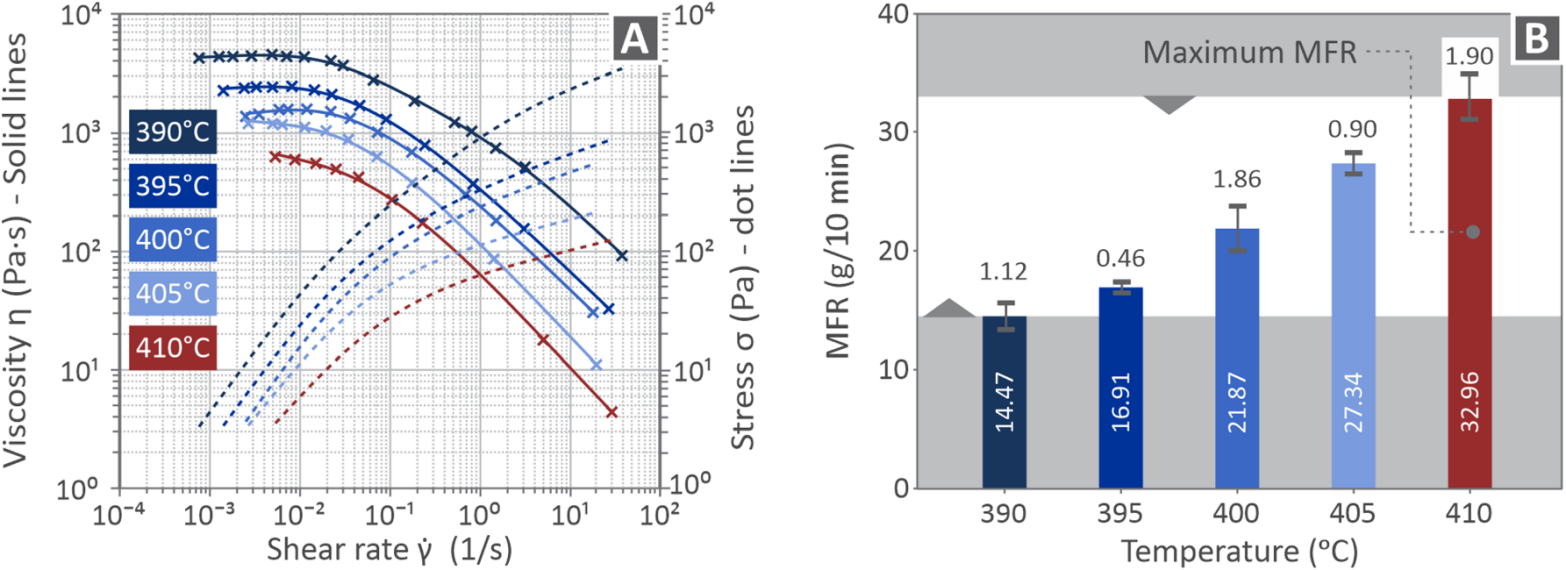
(A) Viscosity and stress curves *vs.* the respective shear rate, (B) MFR bars of their levels depending on the temperature.

Fig. 4 presents the rheological properties of the PI samples in a graph containing the viscosity and stress curves *versus* the corresponding shear rate (Fig. 4A) together with the MFR bars (Fig. 4B). As the viscosity curves decrease, the stress curves increase. Each color corresponds to a different temperature, as shown in Fig. 4A and B.

As shown in Fig. 4A, as the temperature increased, the viscosity decreased across the entire range of shear rates. At 390 °C, PI exhibited the highest viscosity and relatively limited shear-thinning behavior. As the temperature increased to 410 °C, the viscosity significantly decreased, and the material demonstrated a more pronounced shear-thinning behavior. Complementing the viscosity data, the MFR results shown in Fig. 4B highlight a steady increase in the flow rate at higher temperatures. MFR values progress from 14.47 g/10 min at 390 °C to 32.96 g/10 min at 410 °C, confirming improved melt processability and flow at elevated temperatures.

### 3.4. Reference mechanical properties of the 3D printed PI parts

The reference mechanical properties of the 3D printed PI parts, as acquired by the respective tests within the context of the research, are presented in Table 4.

**Table 4:** Average values and deviation for reference mechanical properties of the 3D printed PI parts

Tensile strength	83.18 ± 7.61 MPa	Flexural strength	130.05 ± 22.32 MPa
Tensile modulus of elasticity	679.70 ± 48.57 MPa	Flexural mod. of elasticity	2932.49 ± 494.72 MPa
Tensile toughness	6.37 ± 1.15 MJ m^−3^	Flexural toughness	3.43 ± 0.64 MJ m^−3^
DMA-derived flexural modulus	2611 ± 163 MPa	Impact strength (Charpy)	4.70 ± 0.60 kJ m^−2^
Glass transition temperature	232.89 ± 1.23 °C	Vickers hardness	52.14 ± 8.94 HV
Damping factor at DT_g_	1.49 ± 0.11		

### 3.5. Dynamic mechanical analysis and mechanical responses of PI sample

**Fig. 5:**
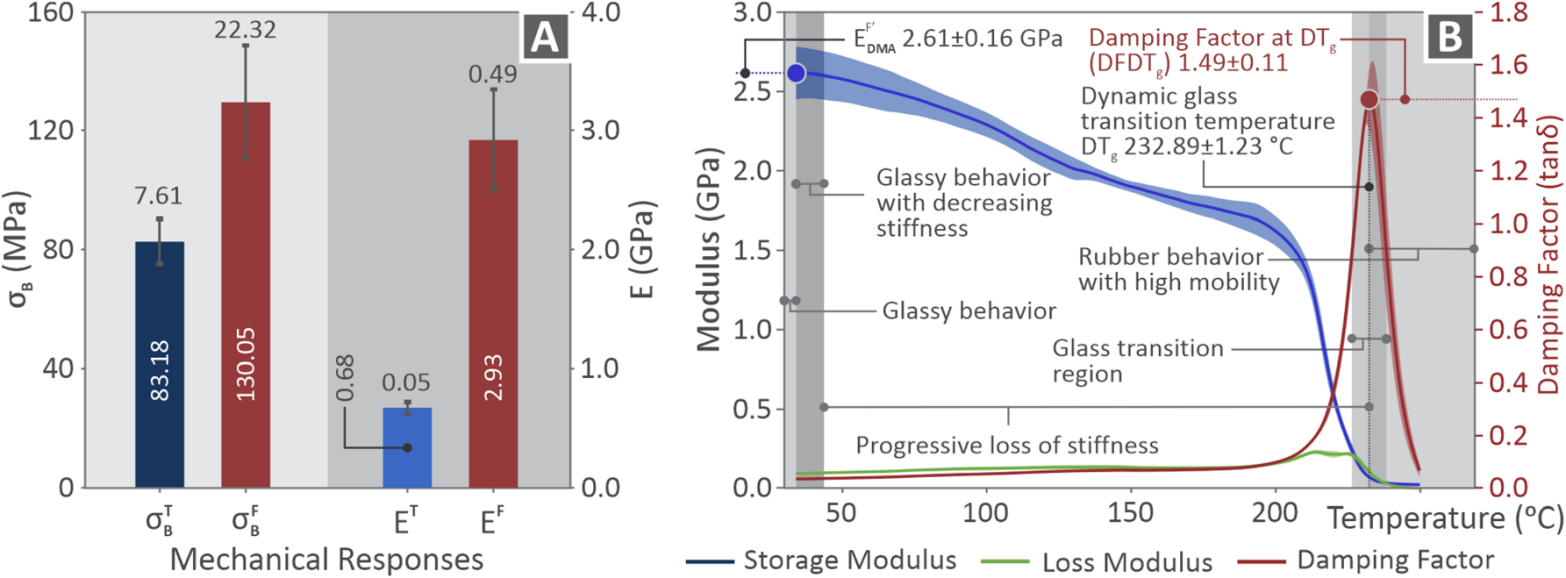
(A) Main tensile and flexural properties for strength and modulus of elasticity corresponded to PI tested with 10 mm min^−1^ elongation speed, (B) DMA curves of the loss modulus in green line, storage modulus in blue line, and damping factor in red line.

In Fig. 5A, the strength and Young's modulus bars indicate the main properties of the PI tested at 10 mm min^−1^ for tensile and flexural characteristics. The DMA results (Fig. 5B) are presented in a graph with curves of the loss modulus (green), storage modulus (blue), and damping factor (red) *versus* temperature. The DMA findings include curves representing the average (bolt lines) and standard deviation (wider colored areas) values. In addition, the flexural storage modulus, damping factor at *T*_g_, and dynamic glass transition temperature were highlighted. The glassy behavior with decreasing stiffness, glass transition region, progressive loss of stiffness, and rubber behavior with high mobility areas are also highlighted in the same figure.

As seen in Fig. 5A, the flexural properties were higher than those of their tensile counterparts. During tensile testing, a uniform uniaxial stress is applied across the entire cross-section of the sample, whereas in flexural testing, a stress gradient is imposed across the entire sample, combining tension on one side with compression on the other, leading to higher values of mechanical properties. Specifically, tensile (*E*_T_) and flexural (*E*_F_) moduli of elasticity are 0.68 GPa and 2.93 GPa, respectively, while tensile and flexural strengths reach 83.18 MPa and 130.05 MPa, respectively.

Fig. 5B illustrates the results of the dynamic mechanical analysis (DMA), highlighting the viscoelastic response of the material across a range of temperatures. The storage modulus 
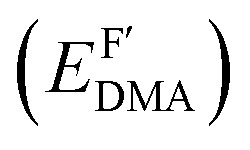
 at low temperatures remains high (2.61 ± 0.16 GPa at ambient temperature), indicating stiff, glassy behavior. As the temperature increased, a gradual decline was observed, leading to a glass transition region. The DT_g_ is identified at 232.89 ± 1.23 °C, marked by a peak in the damping factor (tan *δ* at DFDT_g_), which reaches 1.49 ± 0.11 and reflects the ratio of energy dissipation to energy storage.

### 3.6. Tension results of PI *versus* strain, elongation speed, and strain rate

**Fig. 6:**
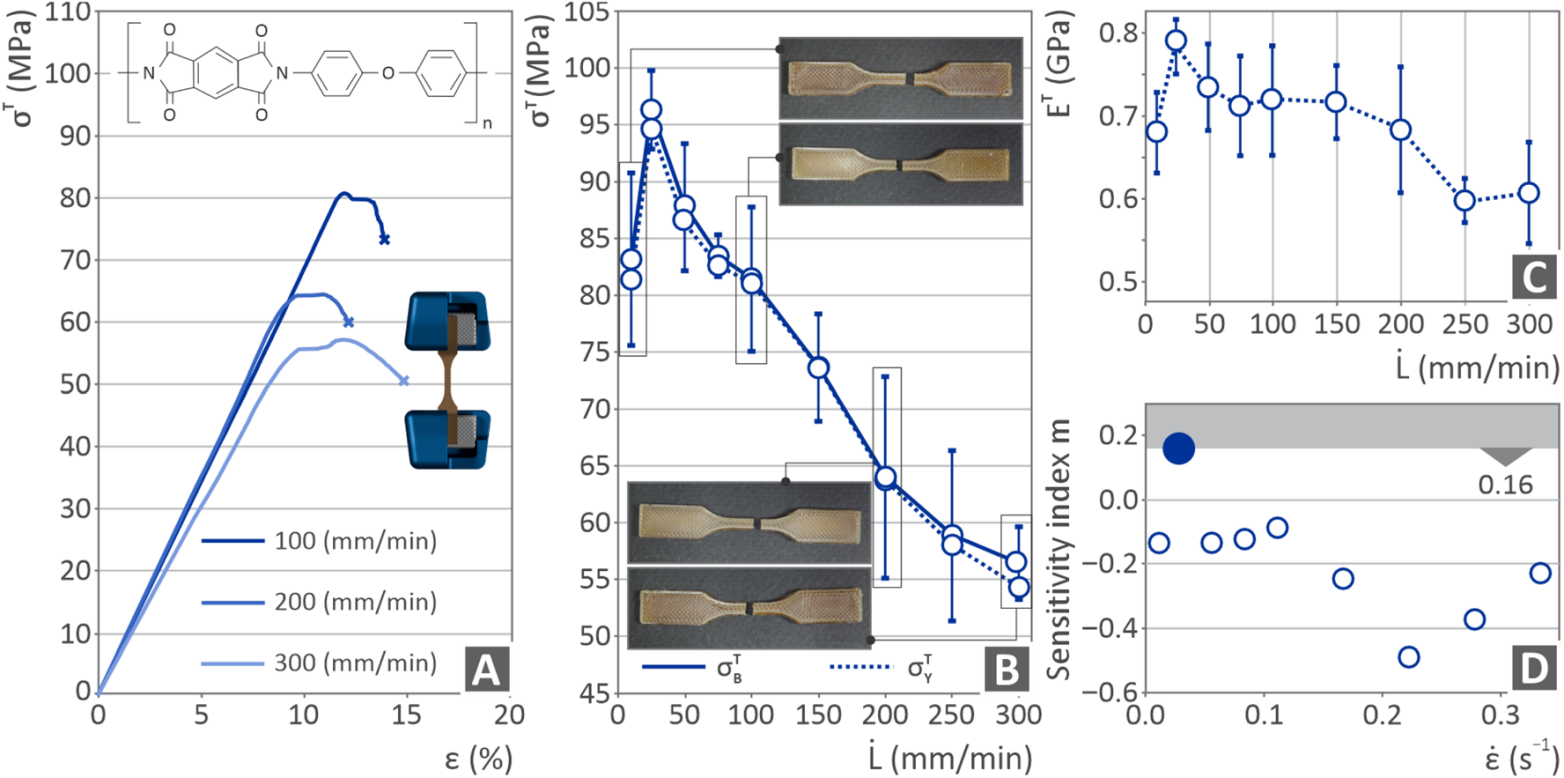
PI tension results including (A) tensile stress as to strain curves for 100, 200 and 300 mm min^−1^ tension speed, illustration of tension testing and PI chemical formula, (B) ultimate strength and yield strength *versus* elongation speed curves in solid and dotted lines, and pictures of tensile tested coupons under 10, 100, 200 and 300 mm min^−1^, (C) tensile modulus *versus* elongation speed results and (D) sensitivity index *m versus* strain rate results.

Fig. 6 shows the results of the tensile mechanical experiments on the PI 3D manufactured coupons. Fig. 6A shows the tensile stress–strain curves for tensile experiments at elongation speeds (strain rates) of 100, 200, and 300 mm min^−1^. Each curve is presented in a different color corresponding to the respective utilized speed. The chemical formula of PI is also included and is placed on the top-left side of the same figure. In Fig. 6B, there are two responses, one represented by the solid line and the other by the dotted line, showing the tensile ultimate and yield strengths *versus* the speed of elongation, respectively. In addition, four images show the coupon samples at testing speeds (elongation) of 10 mm min^−1^, 100 mm min^−1^, 200 mm min^−1^, and 300 mm min^−1^. Both the ultimate and yield strengths increased from 10 mm min^−1^ to 25 mm min^−1^, where the decline began. Fig. 6C depicts the Young's modulus *versus* the speed of elongation results, indicating that there was an increase from 10 mm min^−1^ to 25 mm min^−1^, where the decline began. Fig. 6D shows the sensitivity index metric *m* results *versus* the strain rate, where the highest *m* value was obtained for a 0.028 s^−1^ strain rate of 0.028 s^−1^.

**Fig. 7:**
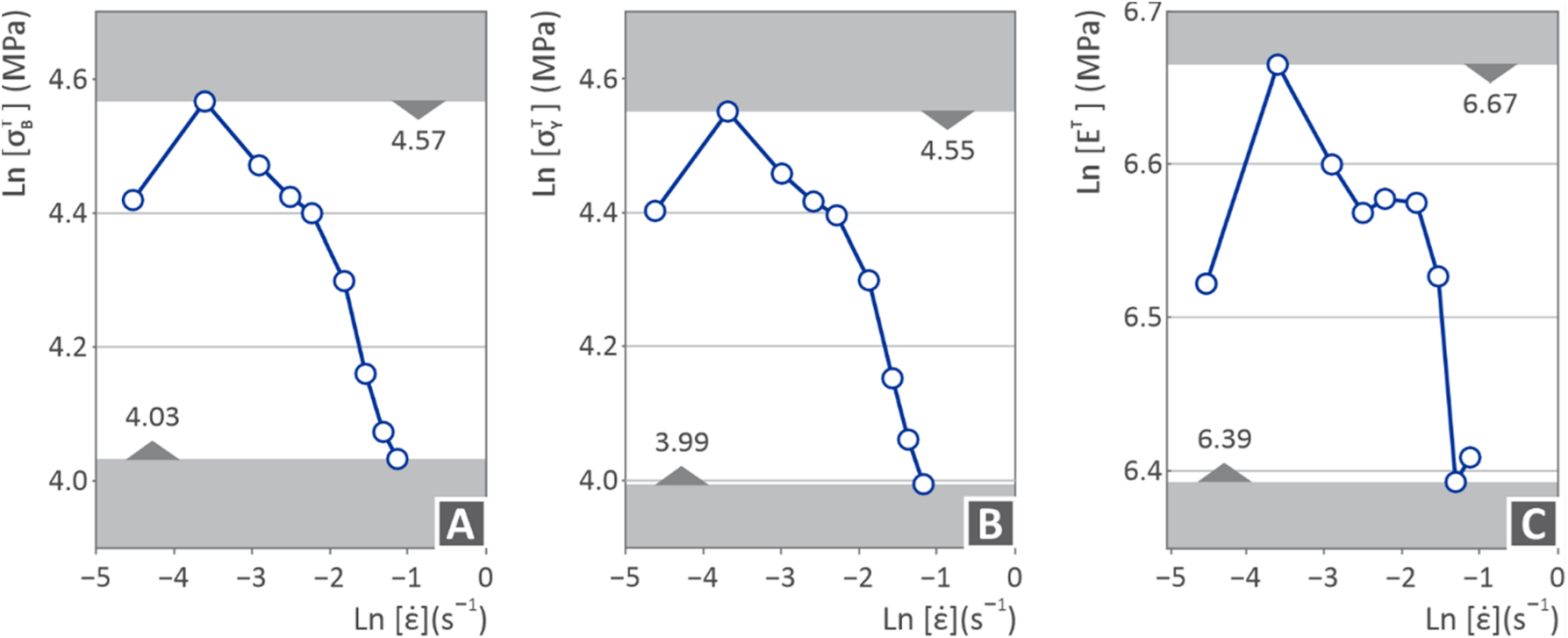
(A) Ln(ultimate strength), (B) ln(yield strength), and (C) ln(Young's modulus), *vs.* the ln(strain rate).

Fig. 7 shows the PI ultimate tensile strength (Fig. 7A), tensile yield strength (Fig. 7B), and tensile modulus (Fig. 7C) (MPa) *versus* tensile strain rate (s^−1^), but on the ln scale. The tensile ultimate and yield strengths seem to increase up to a certain point (from −4.5 to −3.5 strain rate) and then decrease (from −3.5 to −1 strain rate). However, the tensile modulus initially follows the same path (increase from −4.5 to −3.5 strain rate and then decrease from −3.5 to −2.5 strain rate), but at some point, it appears to have a small increase (from −2.5 to −1.5 strain rate) and then continues to decline again (from −1.5 to −1 strain rate).

**Fig. 8:**
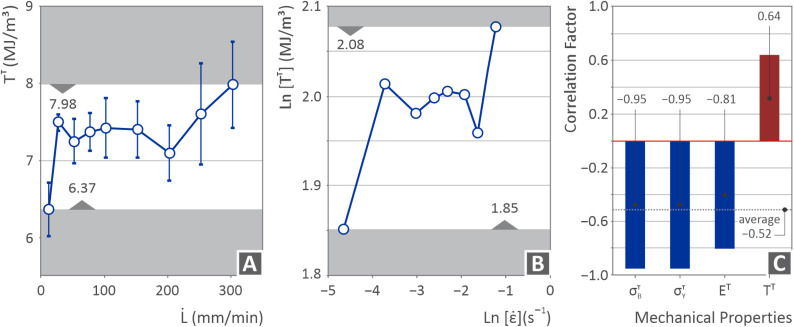
(A) Tensile toughness *vs.* speed of elongation, (B) ln(tensile toughness) *vs.* ln(strain rate), (C) correlation factor *vs.* the examined mechanical properties in this research work.

Fig. 8 shows the PI tensile toughness *vs.* elongation speed (Fig. 8A), tensile toughness *vs.* strain rate on a ln scale (Fig. 8B), and correlation factor *vs.* the mechanical properties examined herein (Fig. 8C). The tensile toughness exhibited a few fluctuations, similar to the ln scale. The correlation factor was calculated in accordance with Pearson's theory.^123^ Positive values denote that the increase in one parameter increases the other, whereas negative values denote the opposite. As shown, positive values were obtained only for the toughness parameter. For the remaining metrics, an increase in the strain rate reduced their values.

### 3.7. Morphological and structural characterization

**Fig. 9:**
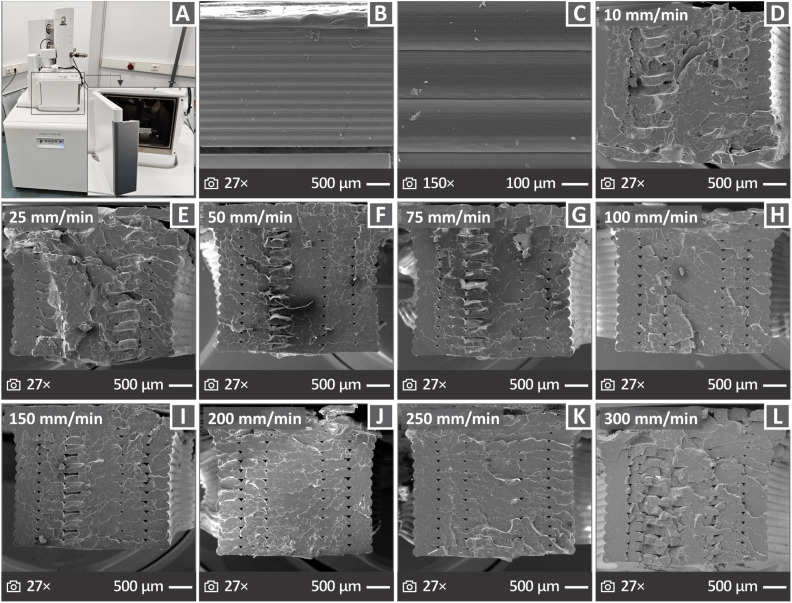
(A) Apparatus for the SEM image capturing, (B) and (C) side surface images in 27× and 150× magnifications, (D)–(L) fractured surface images in 27× magnification from samples derived from all the strain rates tested, respectively.

Fig. 9A shows an image of the SEM apparatus used in this study. Fig. 9B and C presents SEM images of the vertical surfaces of the coupons, magnified at 27× and 150×. In Fig. 9D–L, there are more images from the fractured samples, magnified 27×, for those tested at 10, 25, 50, 75, 100, 150, 200, 250, and 300 mm min^−1^. The images show that the samples with a strain rate of 200 mm min^−1^ exhibited the most brittle behavior. The rest of the samples appear to have more irregular fractured surfaces and defects, while their behavior is not as brittle as that of the 200 mm min^−1^ sample. As far as the side surface images are concerned, they indicate a good distribution of the material, and thus great layering.

**Fig. 10:**
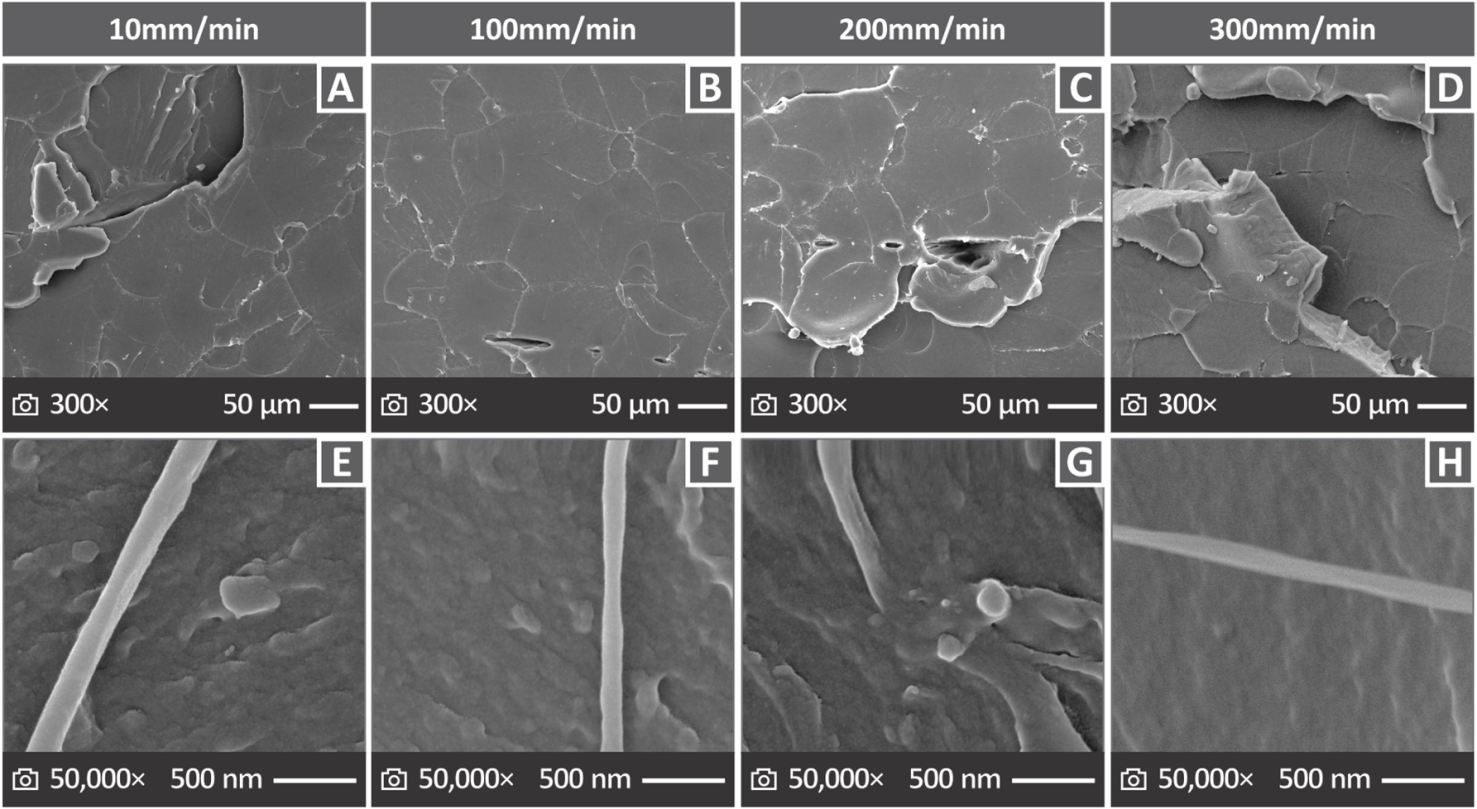
Considering PI tensile coupons assessed with 10, 100, 200, and 300 mm min^−1^: (A)–(D) fractured surface SEM images in 300× magnification and (E)–(H) fractured surface SEM images magnified in 27×.

Fig. 10 presents SEM images of the tensile-tested samples, showing their fractured surfaces. Fig. 10A–D depicts the fractured surfaces at 300× magnification for the samples evaluated at elongation speeds of 10, 100, 200, and 300 mm min^−1^. Fig. 10E–H show the fractured cross sections of the samples at 27× magnification. There are some pores and voids, and brittle behavior appears to characterize them.

## 4. Discussion

This section presents an attempt to interpret and correlate the experimental findings. In the endothermic curve derived through DSC, the relatively sharp transition from a glassy to a rubbery state and the clear signal in the derivative curve suggest a structurally homogeneous material with minimal secondary relaxation. In the cooling cycle (Fig. 3C), *T*_g_ was detected at a slightly lower temperature of 179.9 °C, as seen by the corresponding change in the exothermic heat flow curve and the derivative peak (Fig. 3C, bottom graph). The shift in *T*_g_ upon cooling is typical for high-performance polymers and may be attributed to kinetic factors such as reduced chain mobility and delayed structural relaxation.^124^ Both derivative curves highlight well-defined *T*_g_s with distinct slope changes, confirming the reproducibility and clarity of the thermal event, along with the thermal stability and consistency of the polymer structure, which is crucial for processing, particularly within the extrusion zone temperature range (350–405 °C).

Both DSC and DMA measurements were employed for the glass transition analysis. The findings in the DSC measurements revealed a *T*_g_ temperature of 179.9 °C. The respective *T*_g_ found through DMA was 232.89 °C. The glass transition temperature (*T*_g_) obtained *via* DMA is generally considered to be more accurate than that obtained using DSC. DMA measures the viscoelastic mechanical response of a polymer under oscillatory stress, capturing effects such as the polymer's molecular mobility, which drastically alters its storage modulus, loss modulus, and damping behavior (tan *δ*). In contrast, DSC indirectly measures glass transition by detecting alterations in heat capacity. Through this method, thermal events, such as relaxation or residual crystallization, may affect the findings and are considered in glass transition measurements using the DSC method. Therefore, the DMA *T*_g_ measurement at 232.89 °C should be considered as more accurate. The *T*_g_ value obtained using DMA was significantly higher than that obtained using DSC (∼30% higher). Higher *T*_g_ values are more desirable because they indicate superior thermal stability and mechanical robustness when components operate at elevated temperatures.

The relatively limited shear-thinning behavior, combined with the highest viscosity at 390 °C, is characteristic of restricted chain mobility.^125^ As the temperature increased to 410 °C, the viscosity significantly decreased, and the material demonstrated a more pronounced shear-thinning behavior, reflecting enhanced chain mobility and reduced resistance to flow under shear rate. The corresponding stress profiles (dotted lines) exhibited similar trends, further indicating a consistent response of the polymer melt.

Regarding the rheological measurements, the viscosity graphs show that the viscosity decreases with an increase in temperature. Furthermore, the viscosity decreased with an increase in the applied shear rate, whereas the stress increased. This is the expected behavior, and follows a monotonic pattern. Regarding the MFR measurements, they increased with the increase in temperature as well, having the highest value at the highest temperature of 410 °C tested. The increase in MFR also indicates a decrease in viscosity in this measurement. Therefore, the viscosity and MFR measurements agree, showing a typical polymer behavior, in which the increase in temperature reduces viscosity without any fluctuation in the derived patterns. However, it should be noted that the viscosity and MFR measurements differ significantly. Viscosity is an intrinsic material property that quantifies a polymer's resistance to flow under applied shear stress. It was measured using rheometers across a range of shear rates and temperatures. On the other hand, MFR is a simpler test that determines the mass of polymer extruded through a capillary under a fixed load and temperature over a specified time.

The steady increase in flow rate with higher temperature in the MFR values aligns with the observed decrease in viscosity, as mentioned, because lower melt resistance enables higher throughput under a standardized load. It is evident that PI maintains favorable rheological properties within a specific temperature range, offering a suitable processing window for additive manufacturing applications, where consistent flow and thermal stability are essential. The increase in the MFR values with increasing temperature is a typical viscoelastic behavior of polymers.

As the temperature increases, the thermal energy transferred to the polymer chains is sufficiently high to surpass the intermolecular forces (van der Waals interactions), leading to a decrease in viscosity. This decrease increases the mobility of the macromolecular chains. In 3D printing, this facilitates the flow through the printer nozzle. The aromatic rings located in the PI backbone π–π stacking interactions supported the rigidity and cohesive energy density of the material. When the material is extruded, these interactions are interrupted, thereby enabling a molten flow. Once the material cools, these interactions reform, which enables chain alignment and better adhesion between the layers. The highly polar imide groups (–CO–NH–CO–) generated large dipole moments that attracted other chains. These interactions also occur at elevated temperatures to maintain partial orientation in the structural order of the melt while increasing the strength of the material upon cooling. Weak hydrogen bonding can form between the imide carbonyl oxygens and the residual –NH or aromatic C–H groups. These interactions can alter the melt viscosity and assist in aligning chains during the deposition and solidification (cooling and solid polymer crystalline chain formation) portions of the MEX 3D printing process. MEX 3D printed parts made of PI benefit from the synergistic dependency of the π–π, dipole–dipole interactions, charge transfer, and weak hydrogen bonding interactions that all contribute to the viscoelastic, layer fusion, and final mechanical performance of the PI processed 3D printed parts. The weak bonding and other interactions are partially disrupted and reformed during the printing process, which are all important for high-performance thermoplastic polyimides, to ensure acceptable layer adhesion.

Furthermore, in semi-crystalline or aromatic polymers such as PI, high temperatures can affect physical crosslinking and enhance the flow behavior. The thermal stability should be retained during the process. High temperatures can initiate degradation or chain scission, which may modify the rheological behavior of the polymer and undermine the final mechanical performance of the printed part. The TGA results were used to adjust the nozzle temperature during the 3D printing process to avoid such issues.

In the DMA tests, the sharpness and magnitude of the tan *δ* peak suggested a well-defined transition, indicative of a relatively homogeneous polymer network. This transition denotes the onset of significant chain mobility, and corresponds to a gradual loss in stiffness. The loss modulus remained relatively low throughout the entire temperature range, indicating that the material exhibited minimal damping and high elastic efficiency, specifically at lower temperatures. These results underline PI's capacity of PI to maintain thermomechanical stability over a broad temperature range. While DSC is highly affected by the enthalpic variations associated with the glass transition region, DMA is highly affected by the mechanical relaxation and damping under dynamic loads in this region, acting as a complementary insight to *T*_g_ extracted from DSC.^126^

The tensile test findings presented in Fig. 6, in which the difference in the PI response under different strain rates is presented, show that the strength increased from approximately 82 MPa at 10 mm min^−1^ to approximately 95 MPa at a strain rate of 25 mm min^−1^ (∼14% increase). This is a typical viscoelastic behavior. It should be noted that the 82 MPa tensile strength found at 10 mm min^−1^ is lower than the nominal 92 MPa reported by the manufacturer (10 mm min^−1^ is the strain rate of ASTM D638 used herein and by the manufacturer, as stated in the datasheet) of the material. This difference can be attributed to the structural 3D printing induced in the parts, which lowers the strength of the parts compared to parts made with bulk materials. This difference was expected. For example, for the ABS polymer, 3D printed parts can achieve approximately 80% of the strength of the bulk parts.^127^ Therefore, the manufacturer's claim regarding the ultimate tensile strength of the PI UPP can be verified. A similar pattern can be observed for Young's modulus, with an increase up to a strain rate of 25 mm min^−1^. In this case, the value found at 10 mm min^−1^ strain was significantly lower than the nominal value. This difference can be attributed to the 3D printed structure and the anisotropy induced in the parts. At strain rates higher than 25 mm min^−1^, both the strength and stiffness of the 3D printed parts decrease (the strength decreased by up to 40% at the highest strain rate). Poor interlayer adhesion can contribute to this behavior, which is a common issue in UPP polymers.^128^ As the strain rate increases, insufficient stress redistribution can be achieved, causing internal defects, which act as premature failure initiation sites. Moreover, in certain thermoplastics such as PI, thermomechanical effects can also play a role. A high strain rate can lead to localized heating, which temporarily softens the polymer matrix and reduces its load-bearing capacity.

The toughness (Fig. 8A and B) increased in the samples tested at 25 mm min^−1^ compared to those tested at 10 mm min^−1^. This increase can be attributed to the increase in strength and stiffness of the samples tested at 25 mm min^−1^. Beyond the 25 mm min^−1^ strain rate, it maintained a rather stable value despite the decrease in the strength and stiffness of the samples. The toughness slightly decreased at 200 mm min^−1^ and then started to increase following a rather steep pattern up to the highest strain rate of 300 mm min^−1^ tested in this study. These findings indicate that the samples became more ductile because their strength and stiffness decreased. This was confirmed by examining the fracture region of the 300 mm min^−1^ tested sample (SEM image, Fig. 9L), in which deformation can be observed in contrast to the respective fracture images of the samples tested at lower strain rates, which seem to be more brittle. An increase in toughness with increasing strain rate, despite a decrease in strength, in the PI MEX 3D printed parts can be attributed to the material's energy absorption mechanisms under high strain rates. As the strain rate increased, the polymer chains had less time to align, which delayed brittle crack growth and allowed for more extensive plastic deformation before failure.^129^ Furthermore, the strain rate may have induced thermal softening, leading to locally reduced stiffness, thus allowing greater deformation before failure, particularly in 3D printed structures, which feature stress concentration points at the filament boundaries.^130^ This softening can reduce the strength; however, it may allow the part to deform more before breaking, thereby increasing the material's fracture toughness.^131^ The increase in toughness with increasing strain rate was also confirmed by the calculated correlation factor (Fig. 8C), which had positive values, while it was negative in all other metrics presented in the figure regarding the mechanical properties of the samples.

The strain rate sensitivity index (*m*) (Fig. 6D) is a critical parameter that describes how the flow stress of a material changes with the rate of deformation (strain rate). The strain rate sensitivity index offers valuable insights into defect formation mechanisms. It also provides insight into the mechanical performance of the parts under high-strain-rate loading conditions. A high value of the strain rate sensitivity index indicates a higher capability for energy absorption and, as a result, higher ductility, which is advantageous for applications in which parts are subjected to high strain rate loads. Conversely, lower *m* values signify a more brittle response and reduced time-dependent deformation. A negative *m* value suggests that the strength of the material decreases as the strain rate increases. Thus, the examined parts were more prone to deformation or failure under these conditions. In the MEX 3D-printed PI parts herein, a negative strain rate sensitivity index can denote the strain-softening behavior. Such behavior could have a negative impact on the performance of the 3D-printed components. At a strain rate of 25 mm min^−1^, *m* was positive. This strain rate corresponded at the same time to the best mechanical properties found in the tests. At all other strain rates, the *m*-value was negative, which agrees with the strength values obtained from the experiments (strength reduction).

Table 5 compares the thermal and tensile strength findings reported herein with the respective values for PI parts and other HPP and UPP from the literature. As shown, the tensile strength reported in the current study agrees with the literature for PI UPP. The IDT and *T*_g_ values are lower than those reported in the literature. This can be attributed to the variations in the grades of the tested materials. However, the values are sufficiently high to justify the UPP categorization of the material. *T*_g_ was higher than that of many other polymers in the table. The IDT was lower than that of the other polymers, whereas the tensile strength was approximately average among the materials presented.

**Table 5:** Comparison between the properties of PI and the literature

HPP and UPP	*σ*_B_ (MPa)	IDT (°C)	*T*_g_ (°C)	References
Polyimide (PI)	82	481.8	187.4	
Polyimide (PI) literature	86	573	297	72, 73 and 96
Polyetherimide (PEI)	104	517	208	132 and 133
Polyphenylsulfone (PPSU)	75	525	225	134
Polyetheretherketone (PEEK)	95	510	145	135 and 136
Polyetherketoneketone (PEKK)	84	567	160	137–139
Polyethersulfone (PES)	55	520	191	140 and 141

## 5. Conclusion

High-performance PI thermoplastics for 3D printing were examined in this study. This work presents the variation in tensile properties with increasing strain on MEX 3D printed PI HPPs. The viscoelastic and strain-hardening behaviors of the PI high-performance thermoplastic material, created through additive manufacturing, were investigated and reported. The findings should be considered when designing the respective parts, as in real-life component loadings, which usually have a stochastic nature. Information regarding the optimum extrudability and printability of PI is provided in an effort to form a full guide for this UPP polymer in MEX AM. This by itself has merit considering the high price of the material and the respective available (limited) commercial filaments. The SEM morphological analysis provided interesting illustrations of the lateral and fractured areas of the specimens, showing the impact of different strain rates and revealing an overall brittle response. The results revealed interesting behavior when tested at 25 mm min^−1^, in which the highest strength and stiffness values were obtained (∼95 MPa and ∼0.8 GPa, respectively). As the strain rate increased beyond 25 mm min^−1^, the mechanical properties declined (∼40% lower strength was observed at 300 mm min^−1^). Interestingly, the samples tested at 300 mm min^−1^ exhibited the highest toughness, indicating an increase in the ductility of the PI UPP at this strain rate. Overall, the application of different elongation speeds was proven to affect the performance of the samples, which should be considered when designing parts to be built using the PI UPP with the MEX AM method. Future research can include the investigation of different HPP and UPP, as the findings herein show that this is an area requiring further investigation.

## Author contributions

Nectarios Vidakis: conceptualization, methodology, resources, supervision, project administration; Nikolaos Michailidis: validation, methodology, supervision; Nikolaos Mountakis: validation, visualization, formal analysis; Maria Spyridaki: writing the original draft, investigation; Argyros Apostolos: formal analysis, data curation, visualization; Vasileios Stratiotou Efstratiadis: formal analysis, data curation; Emmanuel Stratakis: supervision, methodology, validation; Markos Petousis: investigation, validation, writing—review, and editing. The manuscript was written with contributions from all authors. All authors approved the final version of the manuscript.

## Conflicts of interest

The authors declare no conflict of interest.

## Supplementary Material

RA-015-D5RA05290D-s001

## Data Availability

The authors confirm that the data supporting the findings of this study are available within the article and its SI. See DOI: https://doi.org/10.1039/d5ra05290d.
