# Supplementary material for: Extrudability, printability, and strain rate sensitivity metrics of lightweight thermoplastic polyimide (PI) in material extrusion (MEX) additive manufacturing
Source: RSC Adv. 2025 Oct 31;15(49):42210–28. doi: 10.1039/d5ra05290d (PMC12577363; doi:10.1039/d5ra05290d)
Supplement: RA-015-D5RA05290D-s001 [file RA-015-D5RA05290D-s001.pdf]

Supplementary Information (SI) for RSC Advances.
This journal is © The Royal Society of Chemistry 2025

Supplementary material for the article:

Extrudability, printability, and strain rate sensitivity metrics of lightweight thermoplastic polyimide (PI) in material extrusion (MEX) additive manufacturing

Nectarios Vidakis [a], Nikolaos Michailidis [b, c], Nikolaos Mountakis [a], Maria Spyridaki [a], Apostolos Argyros [b, c], Vasileios Stratiotou Efstratiadis [b, c], Emmanuel Stratakis [d, e], and Markos Petousis [a*]

[a] Department of Mechanical Engineering, Hellenic Mediterranean University, Heraklion 71410, Greece, vidakis@hmu.gr; mountakis@hmu.gr; mspyridaki@hmu.gr; markospetousis@hmu.gr;

[b] Physical Metallurgy Laboratory, Mechanical Engineering Department, School of Engineering, Aristotle University of Thessaloniki, 54124 Thessaloniki, Greece, nmichail@auth.gr; aargyros@auth.gr; vstratio@meng.auth.gr

[c] Centre for Research & Development of Advanced Materials (CERDAM), Centre for Interdisciplinary Research and Innovation, Balkan Centre, Building B', 10th km Thessaloniki-Thermi road, 57001, Thessaloniki, Greece

[d] Foundation for Research and Technology-Hellas (FO.R.T.H), Heraklion, Crete, Greece, stratak@iesl.forth.gr

[e] Qingdao Innovation and Development Center, Harbin Engineering University, Qingdao, China

[*] Corresponding author: e-mail: markospetousis@hmu.gr (Markos Petousis), Tel.: +302810379227

Abstract

Among the high-performance polymers (HPPs) deployed in material extrusion 3D printing, thermoplastic polyimide (PI or TPI) dominates in terms of chemical stability and thermomechanical performance. Such features, suitable for challenging operational environments, nationalize and defend the remarkably expensive filament market. This study comprehensively explores the physical, thermal, rheological, key mechanical, and strain rate sensitivity metrics of PI. The PI pellets were melt-extruded into filaments under optimized thermomechanical control settings. A detailed experimental course, including elemental and chemical characterizations, Scanning Electron Microscopy morphological assessments, and Raman spectroscopy, wwere implemented. Thermal stability and phase transitions were determined using differential scanning calorimetry and thermogravimetric

analysis. The rheological response was determined through viscosity/stress tests and melt flow rate measurements at various temperatures. A dynamic mechanical analysis was performed. Moreover, standard quasi-static mechanical tests documented the tensile, bending, impact, and microhardness performance of 3D printed specimens. Finally, the strain rate sensitivity metrics of the PI were derived from forty-five 3d printed tensile specimens subjected to nine steps with elongation speeds ranging from 10 to 300 mm/min. Remarkably, the sample tested at 25 mm/min exhibited optimal mechanical performance, whereas a superior toughness was observed at 300 mm/min. The strain rate sensitivity index and various other rate-dependent interactions were determined and comprehensively discussed. The inclusive findings herein provide critical insights into the overall performance of thermoplastic polyimide in additive manufacturing, aiming to support their broader exploitation in advanced engineering applications.

S.1. Raman

A LabRAM HR Raman Spectrometer (HORIBA Scientific, Kyoto, Japan) with a 785 nm laser and 400 mW maximum output was used to acquire Raman spectra. The setup included a grating with 600 grooves/mm, a 400 μm confocal pinhole, and an Olympus (Tokyo, Japan) LMPlanFL N objective lens with 50× magnification at a 10.6 mm working distance. The laser power on the sample was limited to 90 mW using a Neutral Density filter. The measurement volume was 1.7 μm laterally and 2 μm axially, respectively. Raman spectra ranged from 40 to 1900 cm^{-1}, with each point exposed for 2 s and five accumulations. No damage to the sample was observed. Raw data were processed using LabSpec software (HORIBA Scientific, Kyoto, Japan): removing cosmic rays, denoising with a 5-point kernel, background removal with a 5th-grade polynomial, normalization by maximum peak, and cropping between 200 and 1900 cm^{-1}.

S.2. Rheology, EDS, and SEM

A rotational rheometer, DHR-20 rotational rheometer (Discovery Hybrid Rotational Rheometer) (TA Instruments, New Castle, Delaware, United States), was used for rheological examination. The apparatus consisted of two parallel plates and a temperature-controlled environmental test chamber. The rheometer featured a torque range of 1–100,000 μNm and used 25 mm diameter plates with a 1 mm gap. A melt flow index was implemented based on ASTM D1238-13 for the MFR. EDS was performed using a field-emission SEM JSM-IT700HR (Jeol Ltd., Tokyo,

Japan). Morphological evaluation was performed using JSM 6362LV (Jeol Ltd.). (Peabody, Massachusetts, United States) apparatus (mode: high-vacuum, 20 kV, Au sputtering of PI 3D printed coupons).

S.3. TGA and DSC analyses

TGA was performed using an SDT 650 Discovery Simultaneous Thermal Analyzer (TA Instruments, New Castle, Delaware, United States). Indentation was carried out in the temperature range–25-1050°C. These results confirm that the temperatures used for 3D printing did not exceed these levels and caused material degradation. Endothermic and exothermic DSC were conducted with the assistance of a TA Instruments Discovery-Series DSC 25 apparatus (Delaware, USA). A nitrogen atmosphere was used for measurements.

S.4. Mechanical Testing

The aim of this study was to evaluate the effect of the speed at which a load is applied (strain rate) on the performance of a high-performance PI thermoplastic and to report its response. For completeness, mechanical tests were carried out according to the respective standards for uniaxial (tensile, ASTM D638), flexural (three-point-bending, ASTM D790), impact (Charpy, notched, ASTM D6110) loadings, and microhardness (M-H, ASTM D384).

Uniaxial and flexural tests were carried out on an Imada MX2 apparatus (Aichi, Japan) with a test speed of 10 mm/min and 52 mm clearance in the flexural test. Charpy notched impact testing was conducted using a Terco MT220 instrument (Terco, Kungens, Sweden) with a hammer release height of 367 mm. M-H measurements were acquired with an Innova Europe Vickers 300 apparatus, adjusted at 100-gF load, on polished samples, with a 10-second indentation. Five samples were tested in each case under ambient room conditions.

In addition to the static loading tests, Dynamic Mechanical Analysis (DMA) was conducted to evaluate the response of the PI thermoplastic under combined dynamic mechanical (three-point bending) and thermal loading. DMA was performed using a DHR20 Discovery Hybrid Rheometer with a 3-point bending geometry and 40 mm span. The bending samples were 10 mm in width, 50 mm in length, and 3 mm in height. The analysis covered the temperature range of 30–160°C at a heating rate of 5°C/min. During the test, sinusoidal strains ranging from 0 to

0.05% were applied at an oscillation frequency of 1 Hz. The force-tracking system maintained consistent contact with the sample throughout the test.
